# Mitogen-Activated Protein Kinase Expression Profiling Revealed Its Role in Regulating Stress Responses in Potato (*Solanum tuberosum*)

**DOI:** 10.3390/plants10071371

**Published:** 2021-07-05

**Authors:** Madiha Zaynab, Athar Hussain, Yasir Sharif, Mahpara Fatima, Mateen Sajid, Nazia Rehman, Xuewei Yang, Khalid Ali Khan, Hamed A. Ghramh, Shuangfei Li

**Affiliations:** 1 Shenzhen Key Laboratory of Marine Bioresource & Eco-Environmental Sciences, College of Life Sciences and Oceanography, Shenzhen University, Shenzhen 51807, China; madiha.zaynab14@gmail.com (M.Z.); yangxw@szu.edu.cn (X.Y.); 2 Genomics Lab, Department of Life Science, University of Management and Technology (UMT), Lahore 54770, Pakistan; athar.hussain@umt.edu.pk; 3 College of Plant Protection, Fujian Agriculture and Forestry University, Fuzhou 350002, China; 2171904004@fafu.edu.cn; 4 College of Agriculture, Fujian Agriculture and Forestry University, Fuzhou 350002, China; mahparafatima14@gmail.com; 5 Department of Horticulture, Ghazi University, Dera Ghazi Khan 32200, Pakistan; Msajid@gudgk.edu.pk; 6 National Institute of Genomics and Advanced Biotechnology, National Agriculture Research Center, Islamabad 44000, Pakistan; naziarehman@parc.gov.pk; 7 Research Center for Advanced Materials Science(RCAMS), King Khalid University, P.O. Box 9004, Abha 61413, Saudi Arabia; kkhan@kku.edu.sa (K.A.K.); halgramh@kku.edu.sa (H.A.G.); 8 Unit of Bee Research and Honey Production, Faculty of Science, King Khalid University, P.O. Box 9004, Abha 61413, Saudi Arabia; 9 Biology Department, Faculty of Science, King Khalid University, P.O. Box 9004, Abha 61413, Saudi Arabia

**Keywords:** genome-wide, *StMAPKs*, phylogeny, expression, RT-qPCR, TXY motifs

## Abstract

Mitogen-activated protein kinase (MAPK) cascades are the universal signal transduction networks that regulate cell growth and development, hormone signaling, and other environmental stresses. However, their essential contribution to plant tolerance is very little known in the potato (*Solanum tuberosum*) plant. The current study carried out a genome-wide study of *StMAPK* and provided a deep insight using bioinformatics tools. In addition, the relative expression of *StMAPKs* was also assessed in different plant tissues. The similarity search results identified a total of 22 *StMAPK* genes in the potato genome. The sequence alignment also showed conserved motif TEY/TDY in most StMAPKs with conserved docking LHDXXEP sites. The phylogenetic analysis divided all 22 *StMAPK* genes into five groups, i.e., A, B, C, D, and E, showing some common structural motifs. In addition, most of the *StMAPKs* were found in a cluster form at the terminal of chromosomes. The promoter analysis predicted several stress-responsive *Cis*-acting regulatory elements in *StMAPK* genes. Gene duplication under selection pressure also indicated several purifying and positive selections in *StMAPK* genes. In potato, *StMAPK2*, *StMAPK6*, and *StMAPK19* showed a high expression in response to heat stress. Under ABA and IAA treatment, the expression of the total 20 *StMAPK* genes revealed that ABA and IAA played an essential role in this defense process. The expression profiling and real-time qPCR (RT-qPCR) exhibited their high expression in roots and stems compared to leaves. These results deliver primary data for functional analysis and provide reference data for other important crops.

## 1. Introduction

Plants often experience various environmental stresses, including biotic (diseases, insect pests, weeds, etc.) and abiotic stresses (drought, low and high temperature, salinity, etc.) [1,2]. These factors attenuate plant development and growth, which can badly affect plant productivity and yield quality [2]. Therefore, it is important to understand the stress damage and stress response mechanisms in plants to improve their resistance level [3,4]. The signal transduction pathway is verified to be essential in plant growth and stress resistance [5,6]. In eukaryotes, the MAPK cascade is one of the major signal transduction pathways that control several mechanisms, including cell differentiation [7,8], hormonal and maturation transduction [9], development [10], immunization [11], and various biotic and abiotic stresses in plants [12,13].

Over recent years, the investigation on the MAPK cascade has been increased dramatically and has become a hot research topic. MAPKs are considered the first cascade component that is involved in the activation of MAPKs. The S/TXXXXX-S/T (amino acid type denoted by X) signature in the main loop of MAPKK involved in phosphorylation and dephosphorization leads to the downstream trigger in the activation loop (T-loop) via double phosphorylation of the T-X-Y motif [14,15]. Furthermore, the activated MAPK influences the phosphorylation of transcriptional factors and stimulates the signaling components to regulate the downstream target gene expression [16].

The availability of plant genome assemblies provided researchers with raw materials to examine and identify the essential gene families, their functions, and their evolutionary relationships among different plant species. *MAPK* genes have been prompted through physiological and biological significance, and their potential applications are used to improve plant stress tolerance. The *MAPK* genes have been identified and characterized in several land plants, from lower to flowering plants. Furthermore, many genome-wide studies have been conducted and explored the MAPK gene family, e.g., Arabidopsis [11], purple false brome [17], rice [18,19], white mulberry [20], wheat [21], grapevine [22], maize [23], tomato [24], tobacco [25], banana [26], poplar [27], and apple [28].

Potato is an important economic crop and widely used as a food throughout the world. However, as with other plants, potato yield is also vulnerable to biotic and abiotic stresses [29,30]. Until now, very little is known about MAPKs in the potato genome. Therefore, the current study involved a genome-wide screening of MAPKs in the potato genome. In addition, several bioinformatics analyses were also carried out to explore the basic and advanced features of MAPKs, including gene structure, chromosome localization, phylogenetic relations, conserved protein domains, regulatory networks, expression profiles in different tissues, and relative expression through real-time qPCR. The outcomes of this study will provide the base for further functional analysis of potato *MAPKs* and can also contribute to a better understanding of their molecular mechanisms.

## 2. Materials and Methods

### 2.1. Identification of the StMAPKs

The *Solanum tuberosum* genome was downloaded from Phytozome (https://phytozome.jgi.doe.gov/ accessed on 27 June 2021). Arabidopsis MAPK proteins were obtained from the TIAR (http://www.arabidopsis.org/ accessed on 27 June 2021) and Phytozome (https://phytozome.jgi.doe.gov/ accessed on 27 June 2021) and used as the query sequence. A local BLASTp similarity search was carried out to find the MAPKs in the *S. tuberosum* genome. The collected gene sequences were subjected to PfamScan and Batch CDD-NCBI search to validate the presence of MAPK conserved domains. The data redundancy was removed, and the identified genes were used for further analysis. The protein physiochemistry, including isoelectric point (PI) and molecular weight (MW), was forecasted by an online ProtParam tool available at the ExPASy server. The subcellular localization of *S. tuborosum* MAPK genes was predicted through CELLO v2.5 (https://cello.life.nctu.edu.tw/ accessed on 27 June 2021).

### 2.2. Multiple Sequence Alignment and Phylogenetic Analysis

All identified protein sequences were aligned using MUSCLE with 16 iterations. Then, the aligned sequences were used to construct a phylogenetic tree by a maximum likelihood approach with 1000 bootstrap values.

### 2.3. Chromosomal Locations, Synteny, and Selective Pressure Analysis

Information regarding all known *StMAPKs* was retrieved from the PGSC website. TBtools software (https://github.com/CJ-Chen/TBtools accessed on 27 June 2021) was used to map the chromosomal positions. The comparative synteny analysis for the visualization of genome conservation was executed through the Circoletto Tool (tools.bat.infspire.org/circoletto/ accessed on 27 June 2021). Duplicated genes’ coding sequences were arranged with MEGA7 by using the Muscle (codon) method. The synonymous and nonsynonymous substitution rates (Ka: No. of nonsynonymous substitutions per nonsynonymous site, Ks: No. of synonymous substitutions per synonymous site) were calculated with KaKs_Calculator 2.0 software through the MYN method. Further, the divergence time ‘t = Ks/2r’ was calculated through exchange rate r = 2.6 × 10^−9^ [31].

### 2.4. Gene Structure Analysis and Conserved Motif Identification

Gene Structure Display Server identified the MAPK gene family exon–intron characteristics. However, putative MAPK proteins’ conserved motifs were determined by an online MEME server (http://meme-suite.org/ accessed on 27 June 2021) through subsequent parameters: optimum width ranges: 6 to 200; no. of motifs: 20. TBtools software (http://github.com/CJ-Chen/TBtools accessed on 27 June 2021) was used to build the distribution of the motifs.

### 2.5. Cis-Elements Analysis

To estimate the *Cis*-regulatory elements of all potato MAPK family genes, promoter sequences (2000 bp upstream of initiation codon ‘ATG’) were taken out from the *S. tuberosm* genome in a generic file format. The promoter sequences were scanned with Plant *Cis*-acting Regulatory DNA Elements (PlantCARE) http://bioinfermatics.psb.ugent.be/webtools/plantcare/html/ accessed on 27 June 2021.

### 2.6. Expression Analysis of MAPK Genes

To study the expression analysis of *StMAPK* genes, fragments per kilobase million (FPKM) were observed in different tissues. The collected data were compiled according to highly expressed tissues such as roots, stems, and leaves. The FPKM values were used to draw a heatmap with the help of TBtools [32].

### 2.7. Plant Material and Sample Collection

Potato tubers were obtained from the National Agricultural Research Center (NARC), Islamabad, Pakistan, and planted in a glasshouse under optimum conditions at the National Institute for Genomics and Advanced Biotechnology (NIGAB) NARC, Islamabad, Pakistan. After 25 days of post-germination, young leaves, stems, and roots were collected in replicates and stored in liquid nitrogen for RNA extraction.

### 2.8. RNA Extraction and Quantitative Real-Time PCR Analysis

Total RNA from leaves, stems, and roots was extracted through a quick RNA isolation Kit (Huayueyang, Beijing, China) following the manufacturer’s instructions. The quality of tRNA was assessed by gel electrophoresis using 1% agarose gel. The first cDNA strand was prepared from 0.5 µg tRNA, using random primer and reverse transcriptase PCR master mix (Toyobo Co., Ltd., Shanghai, China), ReverTra Ace reverse transcriptase with gDNA remover (TOYOBO FSQ-301, Shanghai, China). The real-time quantitative PCR was performed in a BioRad CFX96 Real-Time PCR Detection System instrument (Bio-Rad Laboratories) with a 20 uL reaction mixture with SYBR^®^ Green Real-Time PCR Master Mix (TOYOBO QPK-210, Shanghai, China) using gene-specific primers. The thermocycler was set according to the given protocol as follows: denaturation at 95 °C for 15 s, annealing at 55 °C for 15 s, and extension at 72 °C for 15 s.

Target gene amplification was monitored with SYBER Green fluorescence in each cycle. In addition, the specificity of qRT-PCR amplification was routinely checked with melting curve analysis. Data were analyzed by the 2^−∆∆Ct^ method [33,34], while results were represented through relative gene expression level. During this analysis, elongation factor 1-alpha was used as a housekeeping gene. For each experimental observation, four technical replicates were conducted. The sequences of all primers used are listed in (Table S1).

## 3. Results

### 3.1. Identification and Physiological Properties of MAPK Genes

The genome-wide identification of *MAPK* genes in potato resulted in the identification of 22 non-redundant *MAPK* genes. Similar genes with different transcripts were not considered in the present study. Although all proteins contain conserved MAPK domains, they showed high diversity in their sequences. The details of all 22 MAPK proteins, including chromosome number, protein length, molecular weight, isoelectric point (PI), and domain organization, are listed in Table 1. The encoded protein lengths ranged from 294 to 613 amino acids; the molecular weight was from 33.48 to 69.709.84 KDa; and the isoelectric point was from 4.97 to 9.4. The sub-cellular localization of 22 MAPK genes showed their abundance in the cytoplasm, followed by the nucleus (Table 1).

### 3.2. Multiple Sequence Alignment and Phylogenetic Analysis of MAPK Genes

**Figure 1:**
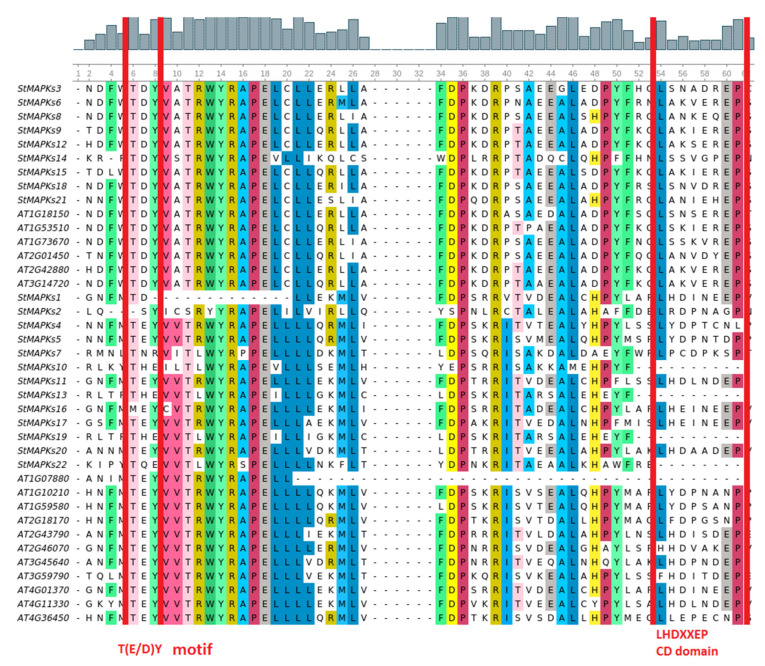
Multiple sequence alignment of AtMAPKs and StMAPKs representing TXY motifs and conserved docking (CD) signatures.

The multiple sequence alignment demonstrated that an important motif, TEY/TDY, was conserved throughout the StMAPKs. However, we also observed little modification in some proteins such as StMAPKs10, StMAPKs13, StMAPKs16, StMAPKs19, and StMAPKs22, with THE, MEY, THE, and TQE. Similarly, the conserved docking site LHEDXXDEP was also observed in most of the StMAPKs with little diversity in some residues (Figure 1).

**Figure 2:**
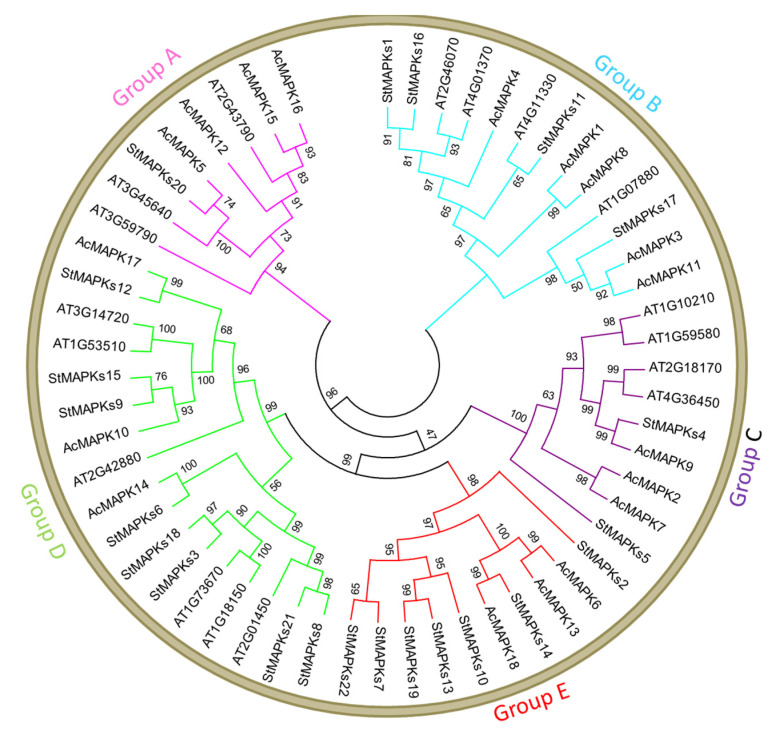
An accumulative phylogenetic tree containing AtMAPK (*Arabidopsis thaliana*), AcMAPK (*Actinidia chinensis*), and StMAPKs (*Solanum tuberosum*). Numbers on the nodes represent bootstrap values.

The combined unrooted phylogenetic tree of *A. thaliana*, Ac, and St divided all MAPKs into five major groups: A, B, C, D, and E. The phylogenetic tree also summarizes the close relationship of St with Ac rather than At by showing a higher number of sister taxa of St-Ac. (Figure 2).

### 3.3. Gene Structure and Conserved Motif Analysis

**Figure 3:**
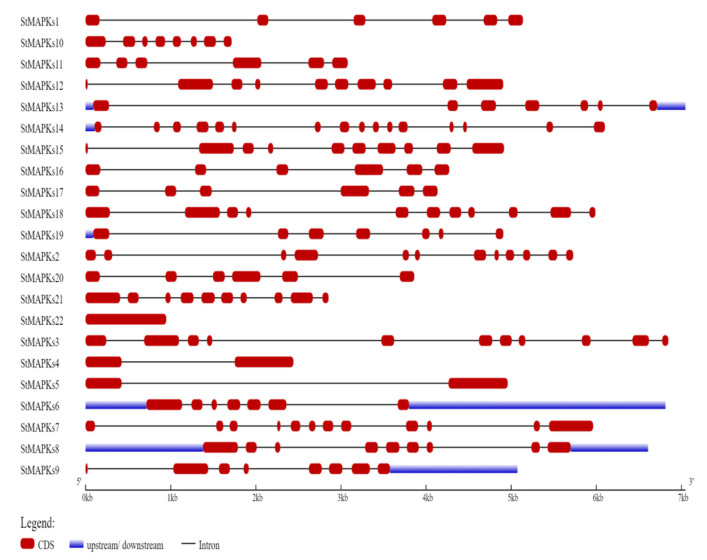
Distribution of exons, introns, and UTR (untranslated regions) in StMAPK gene sequences.

The genomic DNA and coding DNA sequences were used for exon–intron structure analysis in *S.tuborosum*. The number, length, and distribution of exons–introns were not the same among all genes. For instance, *StMAPKs13* was the most extended sequence, and *StMAPKs22* was the shortest among all MAPK genes. The number of *StMAPK* exons ranged from 1 to 16. Of these, *StMAPKs22* has only one exon, while *StMAPKs14* has a maximum of (16) exons. However, some genes such as *StMAPKs1, StMAPKs11*, and *StMAPKs20* have similar exons. In addition, *StMAPKs3* and *StMAPKs18* genes have 11 exons but differ in sequence length (Figure 3).

**Figure 4:**
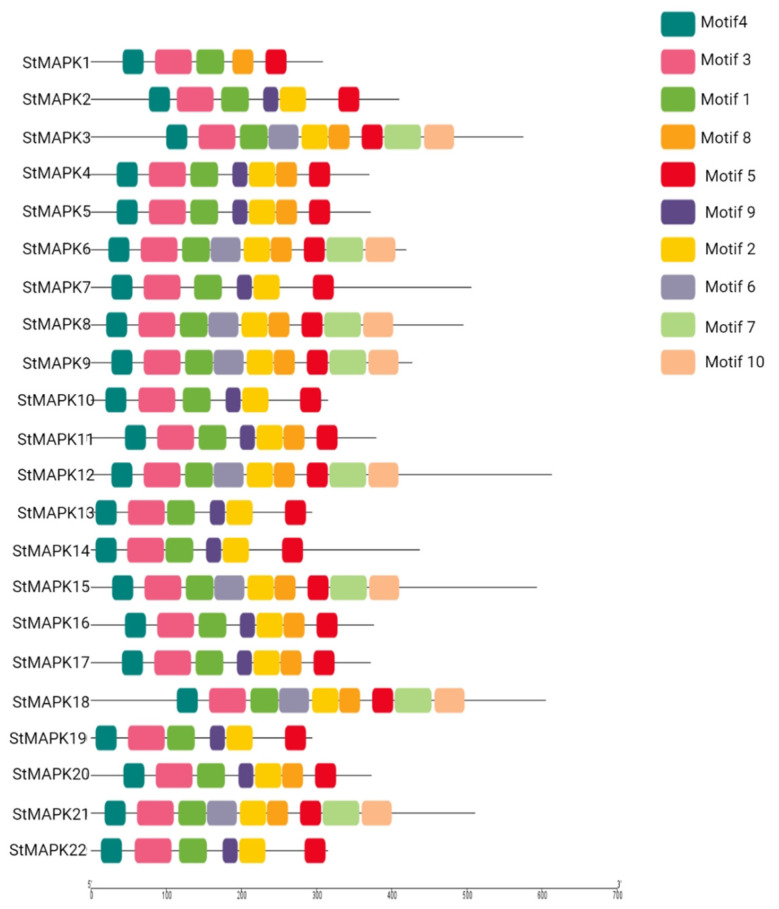
De novo MEME motifs’ distributions in the StMAPK proteins.

The StMAPK proteins’ architecture was also investigated using MAPK amino acid sequences. The MEME motif analysis identified several common and unique motifs in StMAPKs. Commonly shared motifs tended to cluster in the same groups, indicating similar functions [35]. Some motifs including the first, third, fourth, and fifth were observed in all StMAPKs, while the sixth motif was observed in most of the proteins such as *StMAPKs3, StMAPKs6, StMAPKs8, StMAPKs9, StMAPKs12, StMAPKs15, StMAPKs18*, and *StMAPKs21*. Similarly, the seventh motif was conserved in *StMAPKs3, StMAPKs6, StMAPKs8, StMAPKs9, StMAPKs12, StMAPKs15, StMAPKs18*, and *StMAPKs21*. In summary, some motifs were family-specific, some group-specific, some clade-specific, and some taxa-specific. The length of motifs also varied, e.g., the first motif had 38 amino acids (aa), and the third and seventh motifs had 50 aa, while the fourth and fifth motifs had 29 aa (Figure 4).

### 3.4. Chromosomal Distribution of MAPK Genes and Cis-Element Analysis

**Figure 5:**
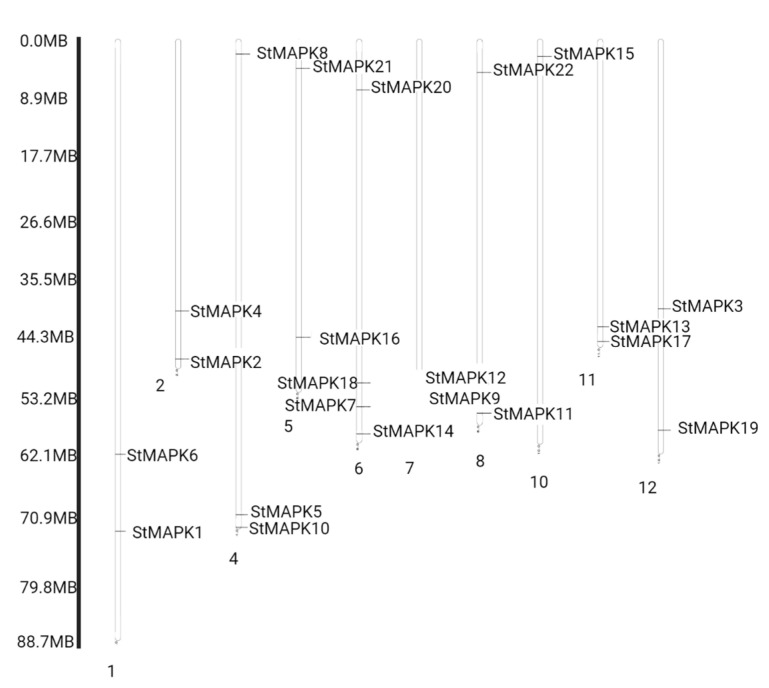
Chromosomal Distribtion of StMAPK genes.

**Figure 6:**
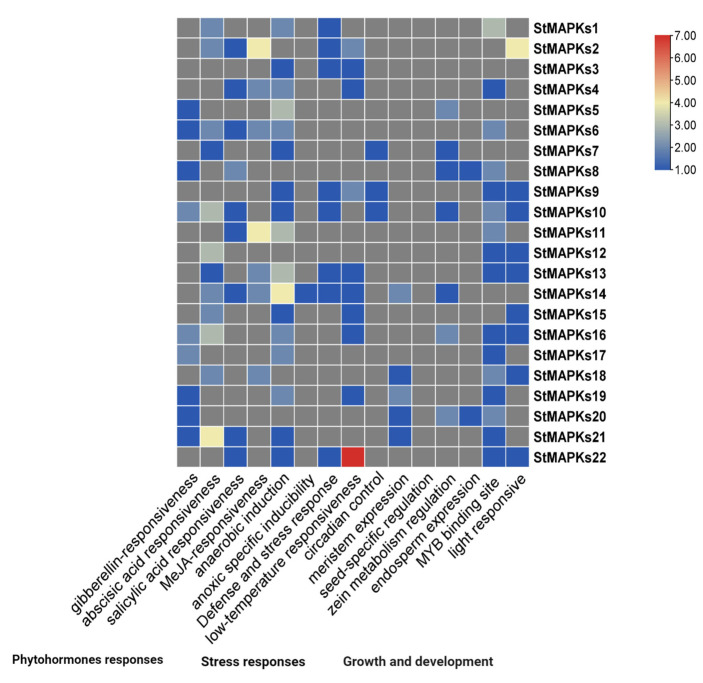
*Cis*-acting elements’ distribution in the regulatory regions of StMAPKs.

The numbers of *StMAPK* genes were arranged in descending order on chromosomes, e.g., chr#6 (four genes), chr#4 (three genes), chr# 1, 2, 5, 7, 8, 11, and 12 (two genes each), and chr#10 (only one gene). However, chr#3 and 9 have no MAPK genes (Figure 5). The *Cis*-element analysis by PlantCARE predicted that most *Cis*-acting sites were from three groups: phytohormones-responsive, growth- and development-related, and stress-responsive [36]. For instance, the MYB binding site was a major element considered a growth- and development-responsive factor and involves light responses. In contrast, among stress response elements, the anaerobic induction- and low temperature-responsive elements were enriched in their promoters. For phytohormones, the ABRE and MeJRE response factors were observed. Consequently, it was observed that MAPK gene expression is carried out by various *Cis*-regulatory elements (Figure 6).

### 3.5. Gene Duplications of MAPK Genes

The molecular evolution rate was calculated by estimating the value of Ka/Ks for each duplicated gene pair. Ka/Ks > 1 was considered as a +ve selection effect, while Ka/Ks < 1 was considered purifying selection, and Ka/Ks = 1 was considered as neutral selection among the duplicated genes [37]. Our results show that most of the MAPK duplicated genes endured purifying selection pressure during the duplication process, implying that the function of duplicated MAPK genes might not change significantly in the succeeding evolutionary process. In addition, the deviation time between pairs of duplicated genes was also estimated. The cosmic majority of MAPK genes presented a Ks value greater than 0.52, while the corresponding deviation time can be more than 100 million years ago (MYA). Fascinatingly, the Ks value of the duplicated gene pair (*StMAPKs2/StMAPKs12*) was 3.77, while the corresponding duplication time may be 119.87 million years ago (MYA) (Table 2).

### 3.6. Comparative Synteny Analysis of Identified MAPK Protein Sequences

**Figure 7:**
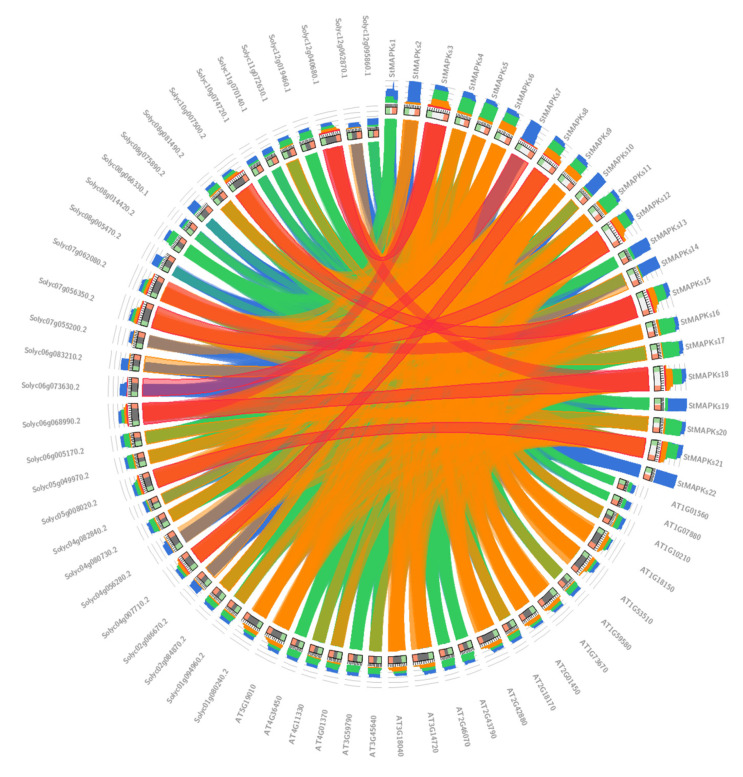
Synteny analysis of MAPKs among *Sl, St*, and *At*.

Comparative synteny analysis among Arabidopsis (*Arabidopsis thaliana*), potato (*Solanum tuberosum*), and tomato (*Solanum lycoperiscum*) demonstrated a remarkable relationship in terms of gene evolution, expression, duplication, triplication, and function. It was noticed that the tomato Solyc08g014420.2 gene sequence illustrated synteny with the potato *StMAPKs20* gene sequence. Similarly, potato gene *StMAPKs18* showed synteny with tomato Solyc06g068990.2. Potato *StMAPKs15* presented synteny with tomato Solyc10g007500.2 (Figure 7).

### 3.7. Tissue-Specific Expression Profiling

**Figure 8:**
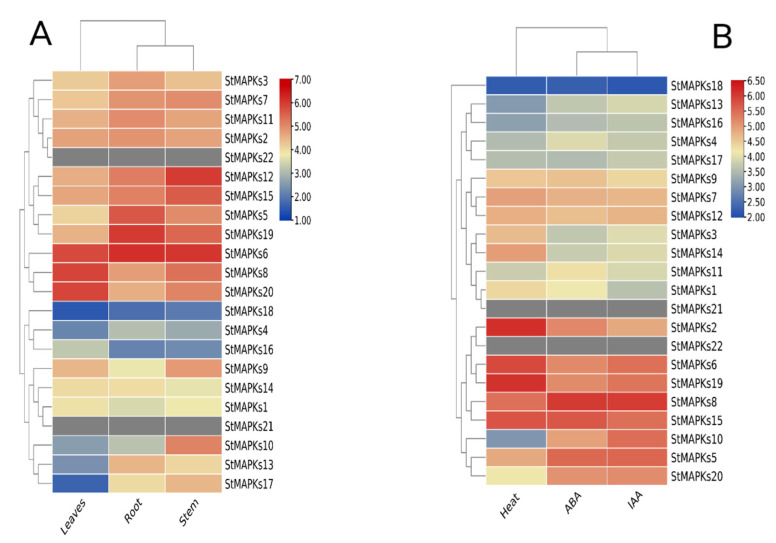
Expression profiling of *StMAPK* genes in (**A**) leaves, roots, and stems (**B**) in response to heat, IAA, and ABA.

The expression profiling results suggest that most of the MAPK genes showed relatively high transcriptional abundance in roots compared to stems and leaves. However, some genes did not show any expression, whereas others showed tissue-specific expression. For instance, *StMAPKs6* showed a higher expression in all three tissues (leaf, root, and stem), while the *StMAPKs19* gene had high transcript abundance in the roots, and the *StMAPKs12* gene was expressed highly in the stem. Similarly, *StMAPKs5* showed a higher expression in roots rather than stems and leaves. In contrast to the majority of the *StMAPK* genes, *StMAPKs20* specifically showed a higher expression in leaves (Figure 8A).

### 3.8. Expression Patterns of StMAPK Genes in Response to Heat Stress

Based on transcriptional levels of *StMAPKs* in potato under heat stress, we inferred that *StMAPKs* might take part in the potato heat stress response. Twenty MAPK genes were expressed in response to heat treatment. Although the transcription levels of *StMAPK2*, *StMAPK6*, and *StMAPK19* are remarkably enhanced in potato after heat treatment (Figure 8B), the expression level of *StMAPK2* was observed to be higher in potato compared to *StMAPK6* and *StMAPK19*. Furthermore, the expression of *StMAPK18* was lower when compared to other genes. The cladogram clusters demonstrated a strong correlation of gene clusters and their expressions under different heat stresses.

### 3.9. Expression Patterns of StMAPK Genes in Response to Phytohormones

Indole acetic acid (IAA) and abscisic acid (ABA) were chosen to investigate the transcriptional responses of *MAPK* genes to hormone treatments. Leaf tissues were treated with ABA, and the expression pattern of all 22 genes was observed. Total 20 *MAPK* genes showed expression against ABA treatment, and the gene *StMAPK8* showed a higher expression. Similarly, all 20 MAPK genes showed expression against IAA in treated leaves, and the gene *StMAPK8* showed a higher expression (Figure.8B). In contrast to heat stresses, there was a strong correlation of gene clusters and their expression under ABA and IAA treatment.

### 3.10. RT-qPCR Analysis

**Figure 9:**
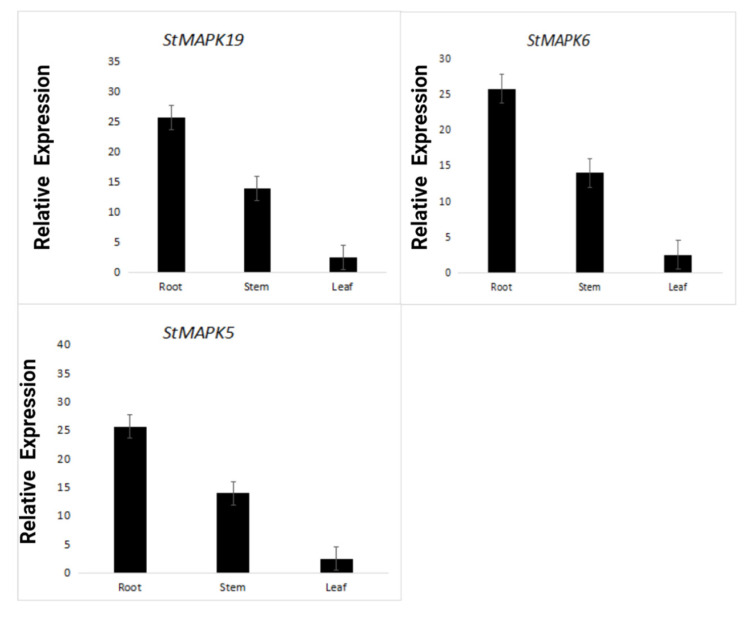
Real-time relative expressions of *StMAPKs* in leaves, roots, and stems.

The RT-qPCR analysis also validated the expression analysis of *StMAPKs5*, *StMAPKs6*, and *StMAPKs19* genes in roots, stems, and leaves. The relative expression pattern demonstrated that all three genes had a higher expression in the roots with two-fold and five-fold changes concerning stems and leaves, respectively. The higher expression in the roots demonstrates their strong association with the soil and environment (Figure 9).

## 4. Discussion

Potatoes are an important component of the world’s food supply [38]. During their growth and development process, potatoes are subjected to a variety of stresses [39]. As a result, the number of MAPK-supporting pathways in plants is increasing on a daily basis, and they are involved in various regulatory processes such as growth and development and response to various biotic and abiotic stresses [40]. Sequence analysis of plant genomes has revealed the presence of MAPK genes in rice [41], poplar [42], *Brachypodium distachyon* [17], and Arabidopsis [43]. However, little information is available for the potato MAPK gene family. In this article, a total of 22 *StMAPK* genes were identified from the potato genome sequence.

All identified StMAPK genes were classified into five groups, e.g., A, B, C, D, and E. Similarly, phylogenetic grouping was also observed in other plants. In general, several conserved regions are present in the protein kinase catalytic domain. For instance, the S/TXXXXX-S/T (amino acid type denoted by X) and T-X-Y motifs have primary structural and functional roles; such catalytic sites and motifs were also conserved in StMAPKs. The sequence similarity of most StMAPK genes around the domains is relatively high. The comparative phylogenetic analysis revealed that the organization of St, Ac, and At proteins was relatively similar amongst each other in groups A, B, C, D, and E, indicating that all *StMAPK* genes in these groups may have descended from a common ancestor.

The structural analysis of StMAPK proteins will be helpful in functional analysis. The evolutionary relic revealed that intron–exon arrangements had shaped the evolution of the gene family [44,45]. This corresponds to earlier scientific findings that some genes tend to be retained in plants that may have short introns or may not have introns during evolution [46]. The expression levels of genes with no and few introns are low in plants [46]. Furthermore, a compact gene structure may allow a rapid expression response to an exogenous or endogenous stimulus [47]. Our gene structure analysis results also suggested that *StMAPK* gene sequences presented the same number of exons–introns with similar functional features because they may have originated during evolutionary processes in the course of duplication events [48,49].

*Cis*-element studies can present an important foundation for additional functional dissection of *StMAPK* genes. We found that all StMAPK promoters contain one or more stress-responsive *Cis*-elements such as LTR (low temperature response element), TATC-box, P-box (MeJA response element), ABRE (ABA response element), and SARE (SA response element). These *Cis*-elements play a vital role in stress response by regulating stress-responsive genes [50]. Therefore, these critical *Cis*-acting sites in the *StMAPKs* suggest their response under various environmental stresses.

The genome size, gene distribution, and duplications in the genome are the main factors of genetic diversity among land plants. The genetic duplication character has long been identified during the origins of gene families’ evolutionary novelty, expression, and complexity. We also found some duplication events in StMAPKs, which may perform a vital role in StMAPK amplification. As gene duplication is an essential feature in the neofunctionalization, expansion, and diversification of gene families [51], similarly, the distribution and mapping of *StMAPK* genes at the chromosome level will help potato breeders to develop new varieties with the desired traits.

It was noted in previous studies that different MAPK members are involved in numerous biological functions such as plant defense, stomata development, pollen development, and different biotic and abiotic stress responses [14,40,52]. Therefore, *StMAPKs’* expression profiling and their validation are helpful for a deep understanding of the potato genome. The current study reported higher transcript abundances of *StMAPKs* in the roots; such evidence was also observed in previous studies [50,52] and supported our results where *StMAPKs19, StMAPKs5*, and *StMAPKs6* show a high expression in root tissues. Our RT-qPCR results show that *StMAPKs19*, *StMAPKs5*, and *StMAPKs6* were specifically upregulated in stem and root tissues compared to the expression in leaves. This suggests that potato MAPK genes have a substantial role in plant growth, development, and response to various stresses. To date, an increasing number of studies have shown that members of the *MAPK* family can play an important role in response to different stresses [53]. In potato, *StMAPK2*, *StMAPK6*, and *StMAPK19* are upregulated in response to heat stress. Hormones can affect the physiological and biochemical reactions of plants through a variety of signal transduction pathways [54,55]. IAA and ABA are important hormones in the plant immune system. A number of studies have revealed that MAPKs are not only involved in stress response but also in developmental and hormonal signaling [56]. To study whether *StMAPKs* in potato were expressed by hormone signaling, potato leaves were treated with IAA and ABA, and gene expression was examined. After IAA and ABA treatment, 20 genes were induced, indicating that different members of *StMAPKs* played different roles in IAA- and ABA-induced defense responses. Under ABA and IAA treatment, the upregulation of 20 *StMAPK* genes revealed that ABA and IAA played an essential role in this defense process, similar to Zhou et al.’s (2017) results [57]. The expression of genes and their clusters also highlighted a strong correlation of gene clusters and their expression under different tissues and stresses. These co-expressions and co-occurrences show their putative role in plant adaptation under diverse environmental stresses.

## 5. Conclusions

Taken together, a total of 22 putative MAPK genes were reported in potatoes. The comparative evolutionary analysis concluded the presence of five major groups in the MAPK family. The conserved structural and functional motifs were present in all StMAPKs, with slight variation among groups and members. The high expression of StMAPKs in roots demonstrated their significant role in plant–soil associations. The presented results provide a deep understanding of major potato plant growth and development challenges under different biotic and abiotic stresses. In potato, *StMAPK2*, *StMAPK6*, and *StMAPK19* showed a high expression in response to heat stress. Under ABA and IAA treatment, the expression of 20 *StMAPK* genes revealed that ABA and IAA played an essential role in this defense process.

## Figures and Tables

**Table 1:** List of identified putative StMAKPs and their features. CDS: coding sequence, pI: isoelectric point.

Gene	CDS	Genomic	Transcript Name	Location	Star-End	No. of Amino Acids	Molecular Weight	pI	Sub-Cellular Localization
StMAPKs1	927	5139	PGSC0003DMT400000192	ST4.03ch01	72545240..72550378	308	35404.2	6.13	Cytoplasmic
StMAPKs2	1233	5729	PGSC0003DMT400001392	ST4.03ch02	47182708..47188436	410	46208.71	8.43	Cytoplasmic
StMAPKs3	1728	6847	PGSC0003DMT400006281	ST4.03ch12	39794625..39801471	575	65699.51	6.47	Nuclear
StMAPKs4	1113	2442	PGSC0003DMT400009066	ST4.03ch02	40117154..40119595	370	42708.53	8.02	Nuclear
StMAPKs5	1119	4960	PGSC0003DMT400009491	ST4.03ch04	70130371..70135330	372	42825.56	6.32	Nuclear
StMAPKs6	1260	3087	PGSC0003DMT400011136	ST4.03ch01	61223835..61226921	419	48384.75	8.37	Cytoplasmic
StMAPKs7	1521	5963	PGSC0003DMT400015241	ST4.03ch06	54220156..54226118	506	56582.89	9.31	Nuclear
StMAPKs8	1488	5700	PGSC0003DMT400015263	ST4.03ch04	2187114..2192813	495	56445.57	6.61	Cytoplasmic
StMAPKs9	1284	3577	PGSC0003DMT400018187	ST4.03ch07	52242577..52246153	427	49320.14	9.03	Cytoplasmic
StMAPKs10	948	1716	PGSC0003DMT400025910	ST4.03ch04	72007479..72009194	315	36055	8.88	Cytoplasmic
StMAPKs11	1140	3080	PGSC0003DMT400031773	ST4.03ch08	55187632..55190711	379	43454.69	5.91	Cytoplasmic
StMAPKs12	1842	4908	PGSC0003DMT400044709	ST4.03ch07	51103717..51108624	613	69709.84	9.25	Nuclear
StMAPKs13	885	6714	PGSC0003DMT400047739	ST4.03ch11	42450013..42456726	294	33480.78	7.09	Cytoplasmic
StMAPKs14	1314	6102	PGSC0003DMT400051979	ST4.03ch06	58205983..58212084	437	49665	5.44	Cytoplasmic
StMAPKs15	1782	4917	PGSC0003DMT400054750	ST4.03ch10	2533056..2537972	593	67184.24	9.22	Nuclear
StMAPKs16	1131	4243	PGSC0003DMT400055751	ST4.03ch05	44001222..44005494	376	43056.15	6.76	Nuclear
StMAPKs17	1119	4136	PGSC0003DMT400065261	ST4.03ch11	44614589..44618724	372	42685.76	4.97	Cytoplasmic
StMAPKs18	1818	5991	PGSC0003DMT400074096	ST4.03ch06	50702397..50708387	605	69042.25	7.07	Nuclear
StMAPKs19	885	5909	PGSC0003DMT400075349	ST4.03ch12	57689402..57694310	294	33688.02	7.12	Cytoplasmic
StMAPKs20	1112	3862	PGSC0003DMT400077272	ST4.03ch06	7463661..7467522	373	42891.19	5.53	Cytoplasmic
StMAPKs21	1536	2855	PGSC0003DMT400078323	ST4.03ch05	4324632..4327486	511	58650.61	6.01	Nuclear
StMAPKs22	948	948	PGSC0003DMT400086876	ST4.03ch08	4905365..4906312	315	36394.48	9.4	Cytoplasmic

**Table 2:** Gene duplication and selection pressure of StMAPKs. Ka: no. of nonsynonymous substitutions per nonsynonymous site, Ks: no. of synonymous substitutions per synonymous site, MYA: million years ago.

Seq_1	Seq_2	Ka	Ks	Ka_Ks	Time (MYA)
StMAPKs3	StMAPKs18	0.1025318	0.5577344	0.1838362	19.717651
StMAPKs9	StMAPKs15	0.0786731	0.5823474	0.1350966	15.129448
StMAPKs8	StMAPKs21	0.1247762	0.5100702	0.2446256	23.995425
StMAPKs4	StMAPKs5	0.1439549	NaN	NaN	27.683632
StMAPKs1	StMAPKs16	0.08686	0.4515929	0.1923414	16.703851
StMAPKs2	StMAPKs12	0.623371	3.7728284	0.1652264	119.87904
StMAPKs13	StMAPKs19	0.0515455	0.5004247	0.1030036	9.9126019

## Data Availability

Not applicable.

## Supplementary Materials

The following are available online at https://www.mdpi.com/article/10.3390/plants10071371/s1, Table S1: Primers used for qRT-PCR.
