# Supplementary material for: Mitogen-Activated Protein Kinase Expression Profiling Revealed Its Role in Regulating Stress Responses in Potato (Solanum tuberosum)
Source: Plants (Basel). 2021 Jul 5;10(7):1371. doi: 10.3390/plants10071371 (PMC8309457; doi:10.3390/plants10071371)
Supplement: Supplementary file 1 [file plants-10-01371-s001.zip › plants-1254559-supplementary.pdf]

Supplementary Table S1

Gene	Forward primer	Reverse primer	Sequence	
StMAPKs6	GGAGGATGTGAGGGAGCTGA	TGACTGCCAGGGACTAACCT	(5'->3')	
StMAPKs5	TGGTTGCATTTTCGCAGAGC	AGAGGCGGGAAAATGGTGTT	(5'->3')	
StMAPKs19	TTCCGGGTGACTCCGAGATT	CTTGCTGGGGTCTAGGCAAA	(5'->3')	
elongation factor 1-alpha	GGCTTGATGACACCAGTT	ACCATACCAGCATCACCGTTCT	(5'->3')	
